# Hecker’s Farina

**Published:** 1874-11

**Authors:** 


### HECKER'S FARINA,

Admirably supplements the White Crushed Wheat. With these two articles of diet and a little fruit, it is possible to live luxuriously, even if nothing else is added. We do not believe in excluding from our tables any of the foods which chemistry shows to be nutritious. Whatever makes good blood and sound muscle, is food, call it by what name you will. But this "Farina" is exceptionally agreeable to all, and "sits lightly" on the stomach—that is, it digests easily, even in the stomachs of invalids and children, whose gastric powers are not as great as those of laboring persons. When cooled it is gelatinous in its character, and dissolves in the stomach far more readily than any animal jelly, while containing much more nutriment.

